# Content of Biogenic Elements in Sheep Wool by the Regions of Slovakia

**DOI:** 10.1007/s12011-024-04328-9

**Published:** 2024-08-08

**Authors:** Martin Janíček, Martin Massányi, Anton Kováčik, Marko Halo, Filip Tirpák, Martyna Blaszczyk-Altman, Marzena Albrycht, Robert Stawarz, Marko Halo, Peter Massányi

**Affiliations:** 1 Institute of Animal Husbandry, Faculty of Agrobiology and Food Resources, Slovak University of Agriculture in Nitra, Tr. A. Hlinku 2, 949 76 Nitra, Slovakia; 2 AgroBioTech Research Centre, Slovak University of Agriculture in Nitra, Tr. A. Hlinku 2, 949 76 Nitra, Slovakia; 3 Institute of Applied Biology, Faculty of Biotechnology and Food Sources, Slovak University of Agriculture in Nitra, Tr. A. Hlinku 2, 949 76 Nitra, Slovakia; 4 Division of Animal Sciences, University of Missouri, S141 ASRC920 East Campus Drive, Columbia, MO USA; 5 Institute of Biology, Faculty of Exact and Natural Sciences, University of the National Education Commission, Ul. Podchorazych 2, 30-084 Cracow, Poland

**Keywords:** Sheep, Wool, Biogenic elements, Pollution, Monitoring

## Abstract

The quality of nutrition and environmental pollution are crucial chemical indicators influencing animal health, reflected in element concentrations in animal tissues and coats. This study investigates the concentrations of biogenic elements (calcium, potassium, magnesium, sodium, copper, and iron) in sheep wool from various regions of Slovakia to compare concentrations and identify possible correlations between individual elements. Samples were collected from six different regions, and concentrations of elements were measured using flame atomic absorption spectrometry. Statistical analysis revealed significant differences in element levels among the examined regions. Calcium concentrations ranged from 729.2 to 4065 ppm, with the highest concentrations in the Kysuce region and the lowest in the Šariš region. Potassium concentrations ranged from 2315 to 3282 ppm, with the lowest values in the Zemplín region and the highest in the Liptov region. Magnesium, sodium, copper, and iron also exhibited varying concentrations across the regions. Correlation analysis identified significant associations between several elements. The findings suggest that regional environmental differences influence element concentrations in sheep wool, highlighting the importance of monitoring biogenic elements for assessing environmental pollution and animal health. Further research is warranted to explore the underlying mechanisms driving element accumulation in wool and its implications for animal welfare and environmental management.

## Introduction

Quality of nutrition and environmental pollution are important chemical indicators when it comes to the health state of animals; these are reflected in element concentrations in animal tissues but also in their coats [1]. Our previous studies were mainly related to concentrations of various biogenic and toxic elements in various biological samples [2–9].

Hair is generally considered a good bioindicator for the state of the environment. Enne [10] observed increased concentrations of toxic elements, especially cadmium, zinc, and lead, in sheep wool from mining regions in Italy. Hair has a minor role in the excretion of minerals [11], though hair physiology is very complex, and more research needs to be done in this field [12]. The hair of animals is essential in biomonitoring the content of trace elements in animals. Certain animals such as badgers have been observed to be high bioaccumulators of metals [13]. As hair is a metabolically inert biological matrix that is chemically homogenous and can be easily collected from domestic animals non-invasively, it is a perfect indicator for the determination of environmental contamination [14]. A common approach nowadays is to assess the chemical exposure of animals to organic pollutants in wildlife but also in farm conditions. It has been found that hair is a useful tool to biomonitor multiple types of compounds, with the important advantage of its non-invasivity [15]. Hair in the follicle is a metabolically active tissue that absorbs and holds minerals in the phase of development, while exposed to circulating blood and body liquids. After it grows above the skin surface and becomes keratinized, it becomes metabolically inactive [16]. Studying elements is important as toxic metals such as Pb, Hg, Cd, As, and Ni can cause neurological, renal, reproductive, and haematological disruptions in organisms, as well as absorption and metabolism of trace elements [17].

The chemical composition of raw wool is highly dependent on breed and environment but can be stated that it consists generally of 33% keratin (which consists of 50% carbon, 12% hydrogen, 10% oxygen, 25% nitrogen, and 3% sulphur) and 26% of dirt, 28% suint, 12% fat, and 1% of mineral water [18]. It is common to find significantly elevated concentrations of heavy metals in sheep wool exposed to environmental pollution [19, 20]. Certain elements, such as Pb and Cd, can change the redox state of cells and promote radical metabolism of cells and tissues which leads to oxidative damage caused by lipid peroxidation [21]. Pollutants show seasonal variation in vegetation, and it was observed that sheep consume vegetation selectively, avoiding the more contaminated plants. Increased clay in the soil can cause increases in cadmium and iron concentrations in the liver and kidney [22]. Worldwide, research is mainly focused on metabolites that could reflect the mineral status. Information on individual feed and mineral intake is limited in groups of animals grazing [23, 24]. The content of elements such as Cu and Zn increases with a longer distance from the skin, meaning it is more exposed to exogenous sources (i.e., soil) and therefore needs to be washed thoroughly [25].

Trace elements significantly affect animal health, reproductive success, and survival. This makes monitoring of their status in populations important for the management and conservation of species [26]. Components of an environment might be burdened by various factors, including industrial pollution and mining waste flowing into freshwater reservoirs. The two adjacent regions—Šariš and Spiš—have both been found to have increased levels of Fe, Cu, Zn, Ni, Pb, and Hg in the environment and generally are the area with the most damaged environment due to centuries lasting mining activities [27]. On the other hand, the Horehronie region is one of the oldest industrial areas in Slovakia and is contaminated by tar dumps by Petrochema Dubová which has been in production for the last 60 years. The main contaminants are Cu, Zn, and Pb in both soil and water sources [28]. Zemplín region is well known to be polluted by 25 years of polychlorinated biphenyls (PCBs) in all environmental components including soil, air, water, and wildlife (especially fish) [29].

Monitoring of the content of toxic elements in environmentally stressed areas due to mining and subsequent processing activities can be observed in many global and European studies. On the territory of the Slovak Republic, one such area is Stredný Spiš, an example of an environmentally burdened area due to long-term mining and industrial activity, which has already ended [30]. Also, our previous clearly reports the relation of risk elements to animal health and reproduction [4, 31–34].

Therefore, the monitoring covered a wide range of regions to assess possible environmental contaminations. The presented study aims to observe concentrations of biogenic elements (calcium, Ca; potassium, K; magnesium, Mg; sodium, Na; copper, Cu; and iron, Fe) in sheep wool from various regions of Slovakia to compare the concentrations and spot possible correlations between individual elements.

## Materials and Methods

### Sample Collection 

**Fig. 1:**
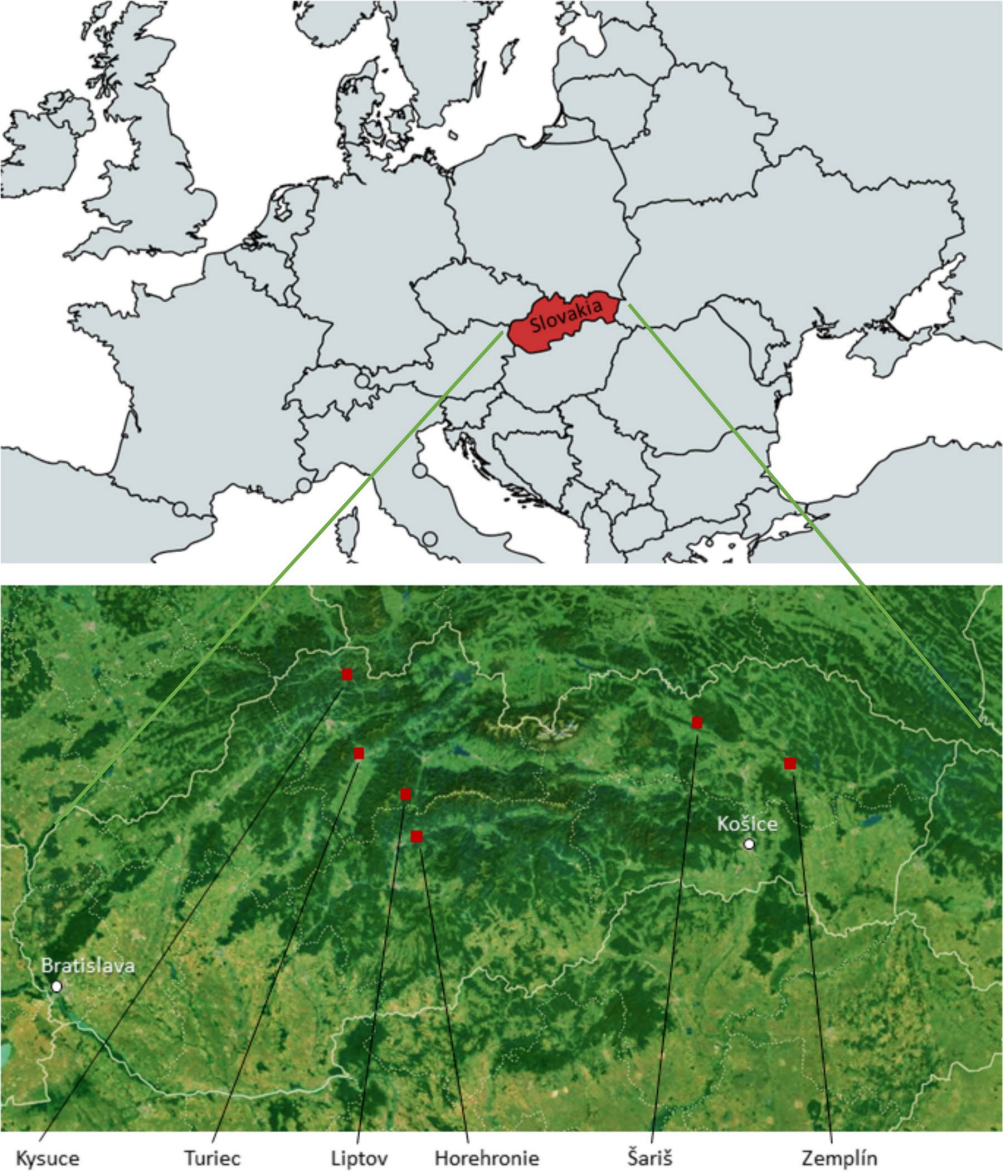
Locations of collected samples in Slovakia; Liptov 48° 55′ 48.8″ N 19° 11′ 51.2″ E; Kysuce 49° 25′ 36.3″ N 18° 50′ 44.1″ E; Zemplín 49° 00′ 37.4″ N 21° 32′ 52.3″ E; Horehronie 48° 46′ 01.8″ N 19° 17′ 16.6″ E; Turiec 49° 05′ 19.4″ N 18° 54′ 56.7″ E; Šariš 49° 12′ 14.6″ N 20° 57′ 03.9″ E

Samples were collected from living animals throughout one summer season from six different regions of Slovakia to represent a comprehensive subject that includes all degrees of possible environmental pollution. Experimental sheep were free to roam grazing and were kept in sheepfold on the pasture. The origins of collected samples were Liptov region, Kysuce region, Zemplín region, Horehronie region, Turiec region, and Šariš region (Fig. 1). Sheep were aged 2 ± 0.5 years. Sheep used in the experiment were consisting of three breeds: Tsigai (Šariš, Turiec, Zemplín), improved Valachian (Liptov, Kysuce), and Lacaune (Horehronie). In total, 106 samples of raw wool were collected, with each sample originating from a different animal. The number of animals in experimental groups was as follows: Liptov 23, Kysuce 18, Zemplín 16, Horehronie 15, Turiec 19, Šariš 15.

### Preparation of the Samples

Dry raw wool samples were collected into polypropylene tubes and stored at room temperature. The wool was washed before the analysis and subsequently air-dried at room temperature. Samples were divided into subsamples directed to metal measurements (weighing 2 g). These were mineralized in an open mineralization system (Velp Scientifica, DK-20) using ultrapure nitric acid (JT Baker, Baker Instra, 65%). The process was carried out with increasing temperature protocol, up to 140 °C; mineralized samples were subsequently diluted up to 10 ml with ultrapure deionized water (18.2 MΩ cm at 25 °C, Direct Q-3, Merck Millipore) [35].

### Analyses of Biogenic Elements

Concentrations of elements (Ca, Cu, Fe, K, Mg, and Na) were measured in mineralized samples with the use of a flame atomic absorption spectrometer (AAnalyst 200, PerkinElmer, USA). Using this method, each measurement consisted of three readings. After every twentieth sample, the quality control solution (QC) was analysed. The entire protocol was compared against the certified reference materials and was compared to the limit of detection (LoD). The final results were expressed as ppm of the dry weight [36].

### Statistical Analysis

GraphPad Prism 8.0.1. (GraphPad Software, San Diego, CA, USA; www.graphpad.com) was used for the statistical analysis. Column statistics were calculated to express means ± standard deviation (SD) of every measured element in all examined regions. One-way ANOVA followed by Tukey’s multiple comparisons test was used to determine significant differences between the element levels of the observed regions. Afterward, Spearman R correlation analysis was used to determine possible associations between the observed biogenic elements. Regression lines were used to visually express the dependencies between statistically significant correlations with the regions as covariates.

## Results

**Fig. 2:**
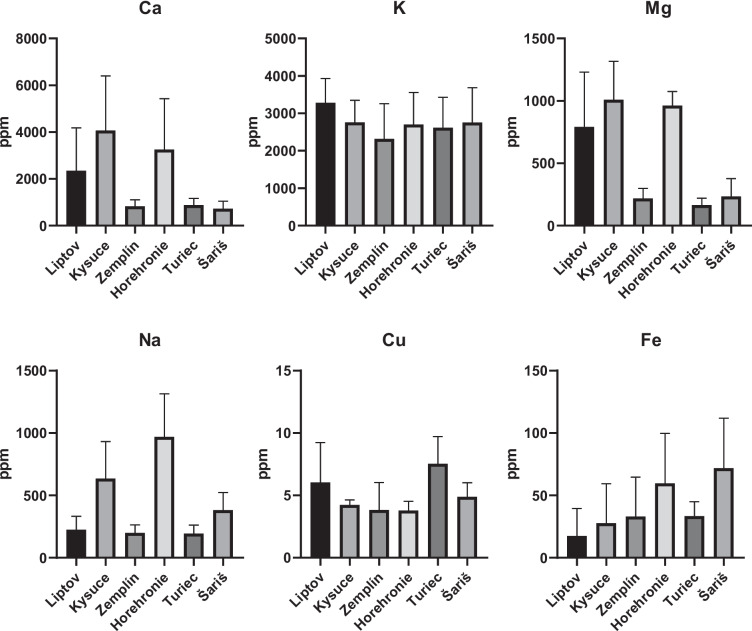
Concentrations of individual biogenic micro and macro elements in sheep wool. Each column represents mean ± SD

Concentrations of various elements examined in the experiment varied between the selected regions. The results are presented in Fig. 2. The average Ca values in the samples ranged from 729.2 to 4065 ppm. The highest concentrations were found in the Kysuce region (4065 ± 2340 ppm), while the lowest were found in the Šariš region (729.2 ± 313.3 ppm). K concentrations were relatively consistent in all samples; the lowest values were recorded in the Zemplín region (2315 ± 940.9 ppm), and the highest were found in the Liptov region (3282 ± 649.7 ppm). The levels of Mg were the highest in the region of Kysuce (1009 ± 308.3 ppm), followed by the region of Horehronie (962.6 ± 113.4 ppm), and the lowest levels of this element were recorded in the region of Turiec (163.8 ± 56.72 ppm). Na levels were highest in the Horehronie region (969.2 ± 344.7 ppm), and lowest in the Turiec region (194.9 ± 65.15 ppm). Cu concentrations were relatively balanced, with the highest average concentrations in the Turiec region (7.54 ± 2.16 ppm), followed by the Liptov region (6.03 ± 3.21 ppm), and the lowest average in the Horehronie region (3.79 ± 0.73 ppm). Fe concentrations were the highest in Šariš (71.83 ± 40.06 ppm) and Horehronie (59.60 ± 40.11 ppm); other locations were comparable, with the lowest Fe content for the Liptov region (17.61 ± 21.92 ppm).

**Fig. 3:**
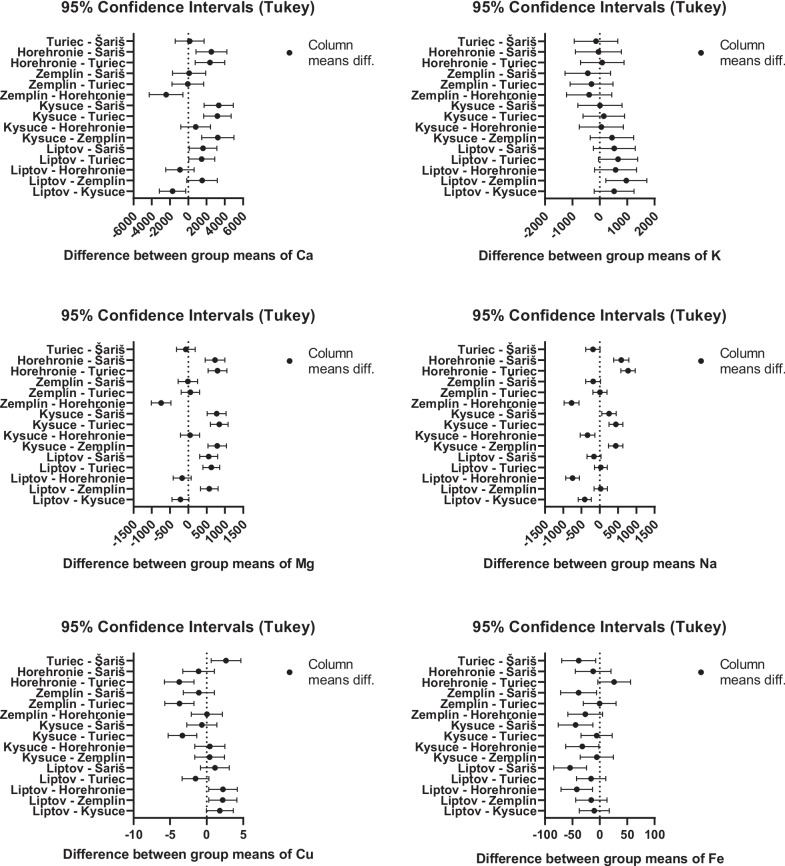
Differences between biogenic micro and macro elements means according to regions presented using 95% confidence intervals (Tukey)

Differences between means of biogenic elements according to regions are presented using 95% confidence intervals (Tukey) in Fig. 3. The results of this analysis compare concentrations of micro and macro elements and express statistically significant differences between individual regions. Confidence intervals that do not contain zero indicate significant differences. The levels of statistical significance were as follows: Ca—Liptov vs. Kysuce (*p* = 0.009), Turiec (*p* = 0.038), and Šariš (*p* = 0.029); Kysuce vs. Zemplín (*p* < 0.001), Turiec (*p* < 0.001), and Šariš (*p* =  < 0.001); Horehronie vs. Turiec (*p* < 0.001), Šariš (*p* < 0.001), and Zemplín (*p* = 0.003); K—basically unanimous, the only significant difference was Liptov vs. Zemplín (*p* = 0.004); Mg—Liptov vs. Zemplín (*p* < 0.001), Turiec (*p* < 0.001), and Šariš (*p* < 0.001); Kysuce vs. Zemplín (*p* < 0.001), Turiec (*p* < 0.001), and Šariš (*p* < 0.001); Horehronie vs. Zemplín (*p* < 0.001), Turiec (*p* < 0.001), and Šariš (*p* < 0.001); Na—Kysuce vs. all other regions (*p* < 0.001, except Šariš with *p* = 0.005); Horehronie vs. all other regions (*p* < 0.001); Cu—Liptov vs. Zemplín (*p* = 0.015) and Horehronie (*p* = 0.015); Turiec vs. Kysuce (*p* < 0.001), Zemplín (*p* < 0.001), Horehronie (*p* < 0.001), and Šariš (*p* = 0.004); Fe—Šariš vs. Liptov (*p* < 0.001), Kysuce (*p* = 0.001), Zemplín (*p* = 0.011), and Turiec (*p* = 0.007); Horehronie vs. Liptov (*p* < 0.001), and Kysuce (*p* = 0.033).

**Fig. 4:**
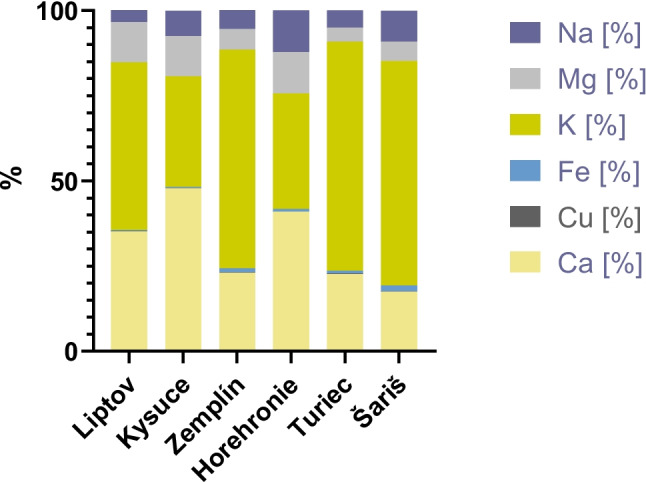
Percentage representation of biogenic micro and macro elements in monitored samples from individual regions

The representation of individual elements (expressed in percent) in the monitored regions is presented in Fig. 4. By region, Liptov (49%), Zemplín (64%), Turiec (67%), and Šariš (66%), the most abundant biogenic element was K, followed by Ca. On the contrary, Ca was the most represented in the Kysuce (48%) and Horehronie (41%) locations, followed by K (32%, resp. 34%). Mg and Na occurred in concentrations as the third or fourth most represented element. The second least represented biogenic element was Fe (ranges from 0.3 to 1.7%), and the least represented was Cu (ranges from 0.05 to 0.2%).

While observing correlations between elements (all samples; *n* = 106), we found several possible associations (Table 1). Significant negative correlations have been found between concentrations of Ca with Cu and Fe (*p* = 0.006 for both). Strong positive correlations have been found between concentrations of Ca with Mg and Na (*p* < 0.001 for both). Mg levels negatively correlated with Cu and Fe and positively correlated with K concentration. Lastly, a statistically significant negative correlation was found between concentrations of Na and Cu, and a statistically significant positive correlation was found between Na and Mg concentrations.

**Table 1:** Correlations (Spearman *R*) between concentrations of individual elements in sheep wool

	Cu	Fe	K	Mg	Na
Ca	− 0.274**	− 0.273**	0.085	0.749***	0.498***
Cu		− 0.025	0.190	− 0.259**	− 0.351***
Fe			− 0.167	− 0.199*	0.162
K				0.260**	0.188
Mg					0.607***

***Correlation is significant at the* p* < 0.001; **correlation is significant at the* p* < 0.01; *correlation is significant at the* p* < 0.05

**Fig. 5:**
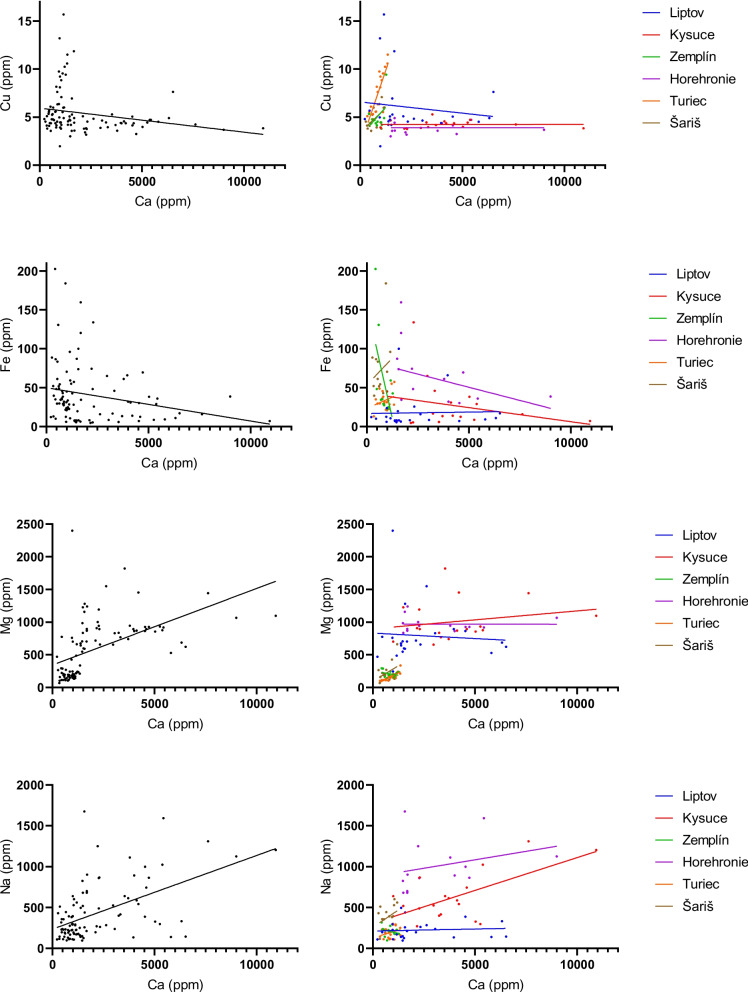
The regression lines for the statistically significant correlations between Ca and other biogenic elements in sheep wool with the regions as covariates (Liptov, blue; Kysuce, red; Zemplín, green; Horehronie, purple; Turiec, orange; Šariš, brown). The straight line represents the best-fit line obtained by linear regression

**Fig. 6:**
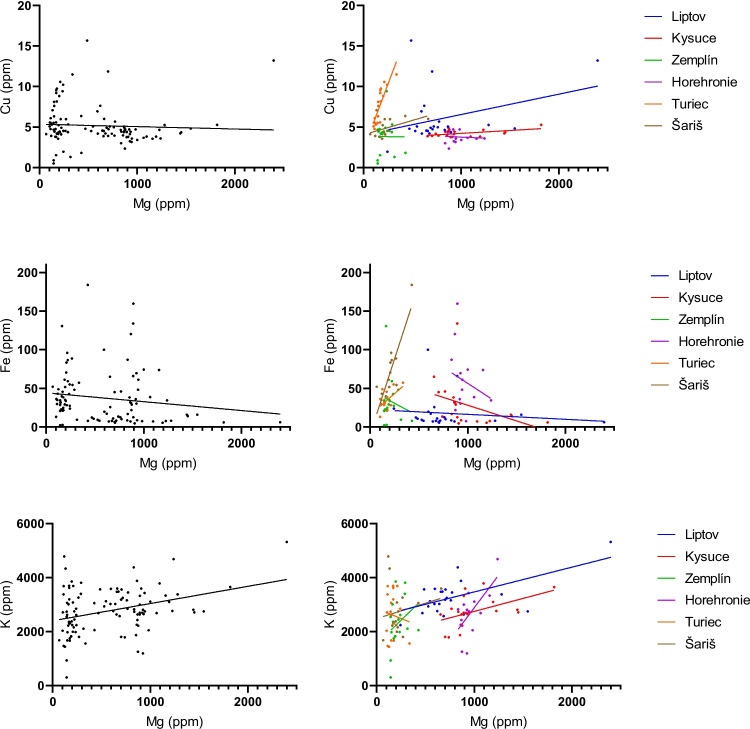
The regression lines for the statistically significant correlations between Mg and other biogenic elements in sheep wool with the regions as covariates (Liptov, blue; Kysuce, red; Zemplín, green; Horehronie, purple; Turiec, orange; Šariš, brown). The straight line represents the best-fit line obtained by linear regression

**Fig. 7:**
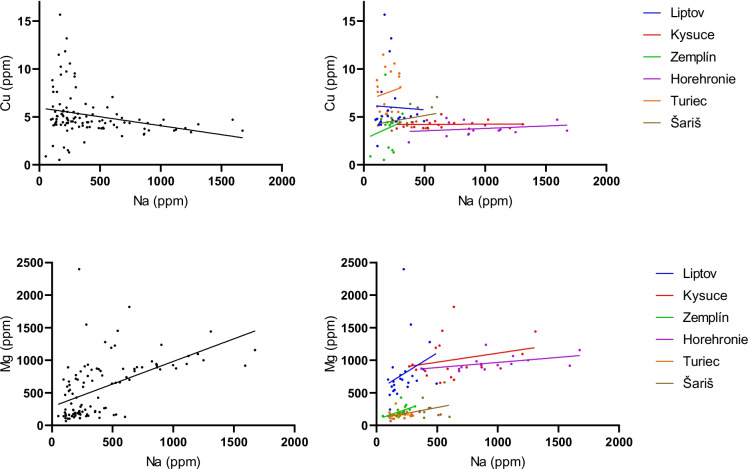
The regression lines for the statistically significant correlations between Na and other biogenic elements in sheep wool, with the regions as covariates (Liptov, blue; Kysuce, red; Zemplín, green; Horehronie, purple; Turiec, orange; Šariš, brown). The straight line represents the best-fit line obtained by linear regression

Regression lines were used to confirm the homogeneity of the observed sample set with the region as a covariate (Figs. 5, 6, 7). Regression lines for Ca to Cu and Ca to Fe associations showed good homogeneity with similar slopes in Liptov, Kysuce, and Horehronie regions. However, the lines for the Zemplín, Turiec, and Šariš regions changed their slope, which may be related to the lower Ca content in the samples from these regions. Regression lines for Ca to Mg and Ca to Na associations showed comparable homogeneity with similar slopes in all regions except the Zemplín region (Fig. 5). Regression lines for the relations between Mg with Cu, Fe, and K are presented in Fig. 6. The relationship of Mg and Cu showed comparable homogeneity in all regions except the Zemplín region, which may be due to lower Mg concentrations in this region. Associations of Mg to Fe and K showed similar homogeneity with similar slopes in Liptov, Kysuce, Zemplín, Horehronie, and partially Šariš region. The region of Turiec showed opposite tendencies, probably influenced by the lowest Mg concentrations in this region. The associations of Na to Cu and Mg showed good comparative homogeneity with similar slopes in all regions (Fig. 7).

## Discussion

Animal hair is essential in biomonitoring the content of trace elements in animals. Certain animals have been observed to be high bioaccumulators of metals [13]. In our study, we monitored a variety of elements of sheep wool across different regions of Slovakia. Hair possesses several characteristics that render it a probable candidate for biopsy tissue. It can be gathered with minimal trauma and stored for analysis at a later, more convenient time, as it does not degrade easily. Moreover, trace elements tend to accumulate in hair at concentrations at least tenfold higher than those found in blood serum and urine [37]. Sheep wool was selected as organic material because of the non-invasive nature of its collection, as well as for the better understanding of element accumulation in it as there is not too much research done on the accumulation of certain elements. The main focus was to observe whether certain environments (especially with high environmental burden) could have a higher effect on element retention in organism.

Patkowska-Sokoła et al. [38] observed chemical content in the sheep wool of different origins—Polish Mountain Sheep, Karagounico Breed, and Awassi breed (Poland, Greece, and Syria)—and breeds and found out that concentrations of elements varied depending on the region of origin. Measured concentrations of Ca ranged between 1790 and 2900 ppm, whereas in our experiment, they ranged between 729 and 4065 ppm. K concentrations ranged from 643 to 755 ppm. Our K results were higher, ranging between 2315 and 3282 ppm. Mg concentrations were much more similar, with their findings ranging between 120.8 and 590.8 ppm, whereas in our experiment, they ranged from 163.8 to 1008.8 ppm. On the other hand, concentrations of Na were much lower in our experiment (195–969.2 ppm) compared to the findings of fellow colleagues, which ranged from 1486.7 to 2165.0 ppm. This confirms that region and breed have a unique effect on concentrations of elements in sheep wool and also indicates that more research needs to be done in the field of element trace research in niche compounds to get a better understanding of values that could be taken as a reference.

Hawkins and Ragnarsdóttir [25] followed the concentrations of certain elements in sheep wool and their changes due to washing related to exogenous contamination. This suggests that the unification of samples is necessary before analysis. In our experiment, we used the same protocol for all samples; therefore, possibility of results being affected by exogenous contamination has been eliminated. They also found correlations between Zn, Cu, Mn, and the age of the animals as well as the colour of the wool.

Another study, conducted on human hair in Poland, described average concentrations of Mg to be 95.9 ppm; Na 241 ppm; K 105 ppm; Fe 24.5 ppm; Cu 23.5 ppm; and Ca 1800 ppm [39]. All concentrations of the elements, except copper, were much higher compared to our results. This suggests that copper accumulation is much higher in sheep wool which may be caused by the worse excretion capability of sheep organisms.

Results of an experiment that studied the hair of infants conducted by Kim et al. [40] support the claim mentioned above with their observed concentrations being Ca 548.1 mg/kg; Fe 6.5 mg/kg; and Cu 0.4 mg/kg. More thorough studies of mechanisms causing the accumulation of certain elements are needed to further understand this problem.

Mg deficiency in ruminants is associated with grass tetany. Cattle suffering from such conditions have been observed to have low concentrations of serum Mg (even below 1.0 mg/100 ml). Hair Mg levels are higher in cattle that were supplemented with Mg [41]. It is important to monitor the concentrations of elements in various tissues and body fluids of animals to control the health state of the animals as it can be a sign of certain diseases. The regulation of plasma Ca ion levels involves active transport within narrow ranges. However, in situations of high Ca intake, Ca is transported passively via paracellular pathways in the jejunum and ileum, regardless of vitamin D intake. In the latter case, Ca absorption likely occurs through passive transport, potentially leading to elevated levels in plasma and wool. The wool’s Ca content may serve as an indicator of dietary Ca intake, particularly in cases of excess [24]. In our experiment, Ca was one of the represented elements out of those measured in the experiment, which indicates high calcium intake by observed animals in all regions and thus its transport to wool.

Similar results in the relationships between Ca and Mg, Ca and Na, and Mg and K were described in sheep milk [42, 43].

In another experiment, the findings were that in each biological substrate measured, including horse hair, the mean Cu concentrations were within the normal range expected in horses. Therefore, they state that bioaccumulation was not observed for this element [44]. This also corresponds with our findings, as measured values were within the range observed by most studies. Also, another research [45] confirms our statements. In their experiment, it was observed that certain elements, such as Ca and K, were significantly increased in sheep milk throughout heavily disturbed areas of Slovakia (especially the Spiš region).

## Conclusion

Our experiment showed visible regional differences between concentrations of individual elements. The concentrations of Ca ranged from 729 ppm in the Šariš region to 4065 ppm in the region of Kysuce. Potassium concentrations ranged from 2315 ppm in the region of Zemplín to 3282 ppm in the Liptov region. Concentrations of Mg ranged from 163.8 to 1009 ppm, with the lowest in the region of Turiec and the highest in the region of Kysuce. A similar range was while observing concentrations of Na from 198.8 ppm (Zemplín) to 969.2 ppm (Horehronie). High Na concentrations are due to environmental pollution caused by Biotika pharmaceutical company in Slovenská Ľupča (Horehronie region) which is in close proximity to the farm where the samples were obtained. Cu concentrations ranged between 3.79 and 7.54 ppm, the lowest concentration in the region of Horehronie and the highest in Turiec. Lastly, Fe ranged between 17.61 ppm in the Liptov region and 71.83 ppm in the Šariš region. Both Šariš and Spiš regions are well-known areas that have a high environmental burden caused by extensive mining activities (including Fe) in the past. Subsequently, multiple associations were found between individual elements based on correlations that highlight their possible synergy and/or antagonism. Based on the results, we can state that there are apparent differences in the environmental statuses of regions in Slovakia, especially coming down to different environmental burdens caused by possible industrial or mining pollution. The positive aspect of this monitoring in this type of matrix is the non-invasiveness of the sampling of biological material. However, by comparing e.g. with blood samples, we can observe high variability between individuals in individual herds. Based on the findings, we can characterize the herd sample or the herd value of the element. In order to reduce the standard deviation in the concentration, we recommend increasing the number of animals in individual groups, as well as supplementing the monitored profile with potentially toxic elements that may represent the level of environmental contamination in both animals and humans.

## Data Availability

The datasets used and analysed during this study were available from the corresponding author upon request.
